# Investigating the Co-Adsorption Behavior of Nucleic-Acid Base (Thymine and Cytosine) and Melamine at Liquid/Solid Interface

**DOI:** 10.1186/s11671-016-1767-0

**Published:** 2016-12-20

**Authors:** Huiling Zhao, Yinli Li, Dong Chen, Bo Liu

**Affiliations:** Institute of Photo-biophysics, School of Physics and Electronics, Henan University, Kaifeng, 475004 People’s Republic of China

**Keywords:** Binary self-assembly, Hetero-compound, Hydrogen binding, Intermolecular recognition

## Abstract

The co-adsorption behavior of nucleic-acid base (thymine; cytosine) and melamine was investigated by scanning tunneling microscopy (STM) technique at liquid/solid (1-octanol/graphite) interface. STM characterization results indicate that phase separation happened after dropping the mixed solution of thymine-melamine onto highly oriented pyrolytic graphite (HOPG) surface, while the hetero-component cluster-like structure was observed when cytosine-melamine binary assembly system is used. From the viewpoints of non-covalent interactions calculated by using density functional theory (DFT) method, the formation mechanisms of these assembled structures were explored in detail. This work will supply a methodology to design the supramolecular assembled structures and the hetero-component materials composed by biological and chemical compound.

**Electronic supplementary material:**

The online version of this article (doi:10.1186/s11671-016-1767-0) contains supplementary material, which is available to authorized users.

## Background

Molecular self-assembly is well-acknowledged as an important bottom-up approach which has been extensively adopted to fabricate nanomaterials under moderate experimental conditions. Presently, there have been various assembled structures fabricated with different dimensions [1–3]. Among them, two-dimensional (2D) supramolecular nanostructures have attracted more attention because of their potential applications. For example, they had been used as primary molecular templates or host for guest molecules in the subsequent multi-component self-assembly systems [4–8]. It had been reported that porous networks formed by 1,3,5-benzenetricarboxylic acid (trimesic acid, TMA) [9–11], 1,3,5-tris(10-carboxydecyloxy)benzene (TCDB) [12–14] supplied the possibilities to immobilize functional guest molecules through matching their native characteristics with each other. Fullerene (C_60_, C_80_) [15–17], metallophthalocyanines (MPc) [12, 13, 16], coronene [17, 18], and metallic ions [19], were introduced and trapped by their favorite networks based on site or size-selective and shape-responsive adsorption effects. Rafael et al. [20] had reported that the honeycomb network composed of perylene-3,4,9,10-tetracarboxylic-3,4,9,10-diimide (PTCDI) and melamine can be filled with ASH, C_12_SH, and BP_3_SH on Au(111)/mica substrate. Wang’s group had also found that binary co-adsorption network formed by a symmetric triphenylene derivative with three carboxyl groups (sym-TTT) and melamine could recognize and catch Fe^3+^ ions efficiently [19].

**Fig. 1:**
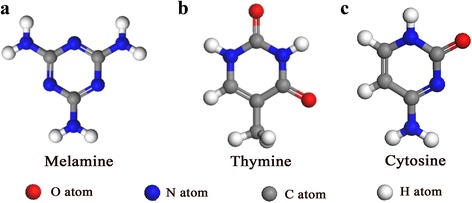
The chemical structures of building blocks. **a** Melamine. **b** Thymine. **c** Cytosine

Generally, the appropriate choice of building block is one of primary fundamental conditions in order to construct an ideal porous network through using molecular self-assembly approach. Among the masses of molecules, melamine (also namely 1,3,4-triazine-2,4,6-triamine, Fig. 1a) was extensively employed as building block in many molecular assembly systems. Possessing a planar molecular structure with three-fold symmetry and three functional groups at the apexes [21–23], melamine tends to form different artistic architectures such as honeycomb network, rosette, and ribbon under ultra-high vacuum (UHV) or ambient condition [20, 24–28]. Meanwhile, melamine-based multiple complexes such as cyanuric acid-melamine were synthesized with ordered and hollow structures, and further fabricated as one class of carbon nitride materials with photo-catalysis property [29]. Except of melamine, nucleic-acid bases including guanine, cytosine, adenine, and thymine, are another one category of unique biomolecules which have incomparable application in biological and gene engineering fields. Their various assembled structures had been fabricated and designed under different experimental conditions in the past decades [30–34]. For example, Flemming et al. had systematically studied the coexistence phenomenon of homo-chiral and hetero-chiral nanostructures of adenine [32], the co-adsorption behavior of guanine-cytosine [30], and the novel quartet-shape of adenine-thymine binary assembly system [33] by using ambient STM technique. Moreover, they had investigated the coordination networks of guanine-potassium (G-K) [34] and guanine-ferrum (G-quartet-Fe) [35] by employing UHV-STM technique. All these works suggest that, melamine and nucleic-acid bases can both act as desirable component during the fabrication of biological materials and the design of molecular devices.

In this work, the co-assembly behavior of melamine and two kinds of nucleic-acid base (thymine, cytosine) was investigated by STM technique at liquid/solid interface. A series of STM results indicated that, the porous construct formed by melamine could not efficiently act as host network to capture thymine or cytosine, although the rigid porous nanostructure of melamine was easily assembled and detected on graphite surface. Actually, phase separation happened during the co-assembly of thymine-melamine binary system, and hetero-component molecular cluster was found in the melamine-cytosine binary assembly system. Combined with geometric optimization of their tentative molecular models, the formation mechanism of as-observed assembled structure was analyzed from the viewpoints of molecular interactions in order to gain insight into the nature of these molecular self-assembly behavior.

## Methods

### Materials

The chemical structures of melamine, thymine, and cytosine are given in Fig. 1. The compounds of melamine (99% purity), thymine (≥99% purity), cytosine (≥99% purity), and the solvent of 1-octanol (99.5% purity) were purchased from Sigma-Aldrich company. They were used without further purification. Three kinds of molecule powder were separately dissolved into 1-octanol solvent with saturated concentration.

### Characterization Technique

All STM experiments were carried out by using Agilent 5500 SPM system (Agilent Technologies, USA) under ambient condition. STM tips were mechanically cut from a piece of Pt/Ir (80/20%) wire with the diameter of 0.25 mm. After dropping molecular solution onto a freshly cleaved 0.25 × 0.25 cm-HOPG substrate (grades ZYA and ZYB, Advanced Ceramics Inc.), a series of STM images were recorded under constant current mode. The specific tunneling conditions were given in the corresponding figure captions.

### Theoretical Calculation

Configuration optimization of molecular model for the self-assembled structures was performed in gas phase by using DMol3 module of Materials Studio software. Generalized gradient approximation (GGA)/PW91 [36] was chosen for exchange-correlation energy, and the calculation accuracy was set up within an appropriate energy cut-off of 280 eV with 2 × 2 × 2 k-point required for these calculations. Considering the performance of the employed computational system, the graphite substrate was not included in all molecular models.

## Results

### Self-Assembly of Melamine-Monomer

**Fig. 2:**
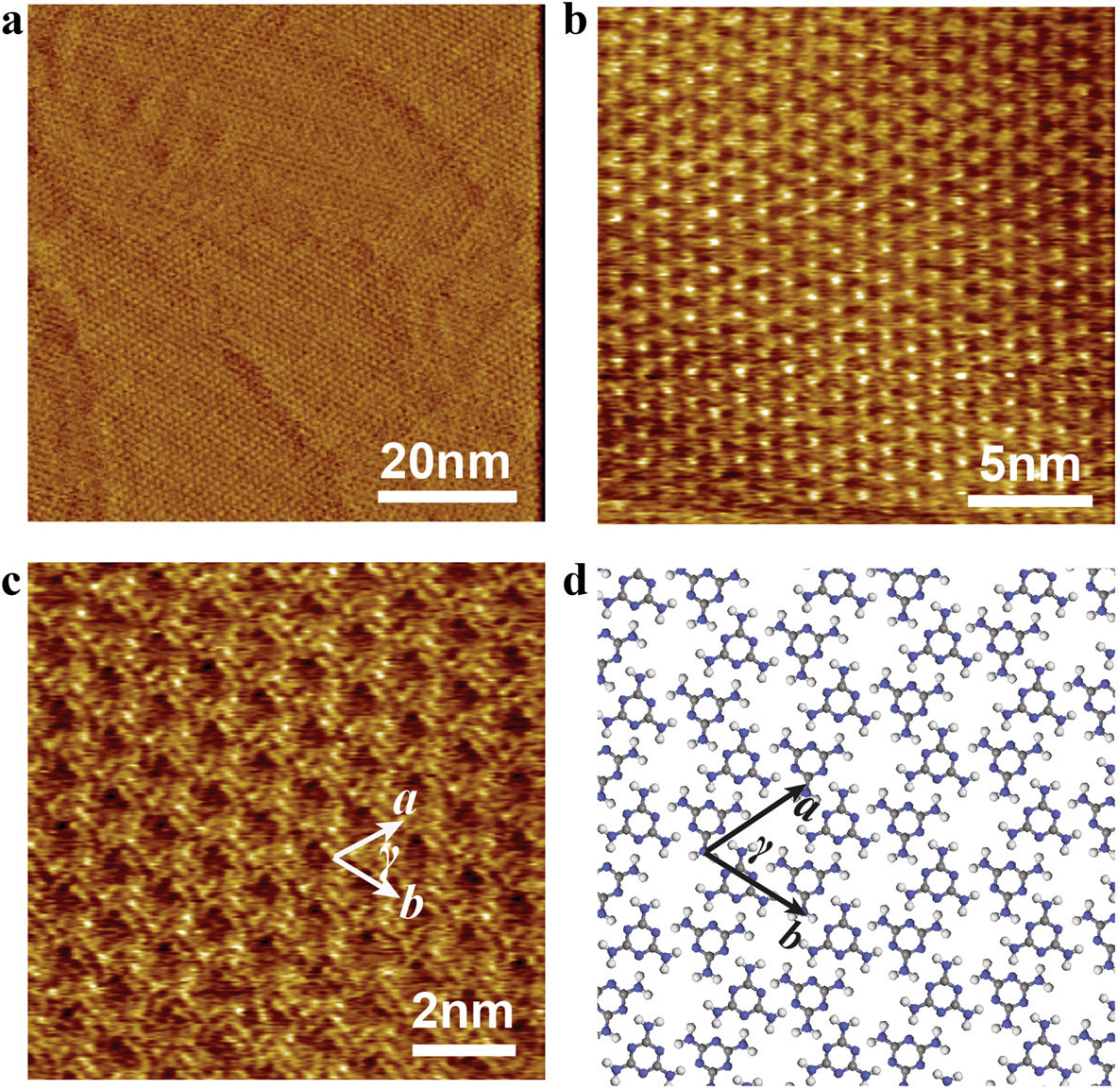
**a** STM image with a large area of the melamine self-assembled structure on HOPG substrate. **b** and **c** High-resolution STM (HR-STM) images for an ordered domain, showing hexagonal porous structure formed by melamine molecules. Tunneling conditions: *V*_bias_ = 0.450 V, *I*_*t*_ = 0.127 nA. **d** Molecular model proposed for the assembled structure formed by melamine molecules

In our experiments, the self-assembly of pure melamine was firstly characterized and used as control experiment for the following sections of thymine-melamine and cytosine-melamine binary assembly systems. A typical STM image for the assembled structure formed by melamine molecules had been given in Fig. 2a, which indicates that the assembled layer with well-ordered pattern had been constructed in a large area after dropping melamine-saturated solution onto a graphite surface. High-resolution STM (HR-STM) images of Fig. 2b and c, reveal that the assembled layer was composed of porous network with hexagonal unit cell which is the characteristic assembled structure of melamine molecules. The diameter of hexagonal unit is ~2.5 nm, which is consistent with those observed under ambient or UHV conditions [20, 23, 27, 37]. According to the molecular feature of melamine possessing planar three-fold (*C*_*3*_) symmetry, the tentative molecular model was proposed and optimized by using DMol3 package of Materials Studio software. The optimized model given in Fig. 2d, shows that one melamine molecule has three nearest neighboring molecules and is fixed by six hydrogen bonds of N···H−N with the bonding distance of ~0.2 nm. Therefore, the lattice unit of the assembled structure formed by melamine molecules has the parameters of *a* = *b* = 1.2 ± 0.2 nm and *γ* = 60 ± 2°, which were outlined in Fig. 1c, d.

Combining Fig. 2c with Fig. 2d, it is obvious that six melamine molecules are linked together to construct a ring-like shape with hole at the center which is the origin of porous network in the assembled monolayer of melamine molecules. Since the assembled structure of melamine possesses homogeneous holes with uniform diameter, this porous network might act as host template to capture some appropriate guest molecule or ions. However, the final products of host-guest reaction are mainly determined by the competition procedure of multiple kinds of intermolecular non-covalent/covalent interactions as well as structure matching features of the host template/network and the introduced guest components under corresponding experimental conditions. With the aim of fabricating well-aligned biomolecular arrays of nucleic-acid bases meshed by the porous network of melamine, two kinds of nucleic-acid bases (thymine; cytosine) were tentatively chosen as guest molecules to investigate their co-assembly behavior at liquid-solid interface.

### Co-Assembly of Thymine-Melamine Binary System

**Fig. 3:**
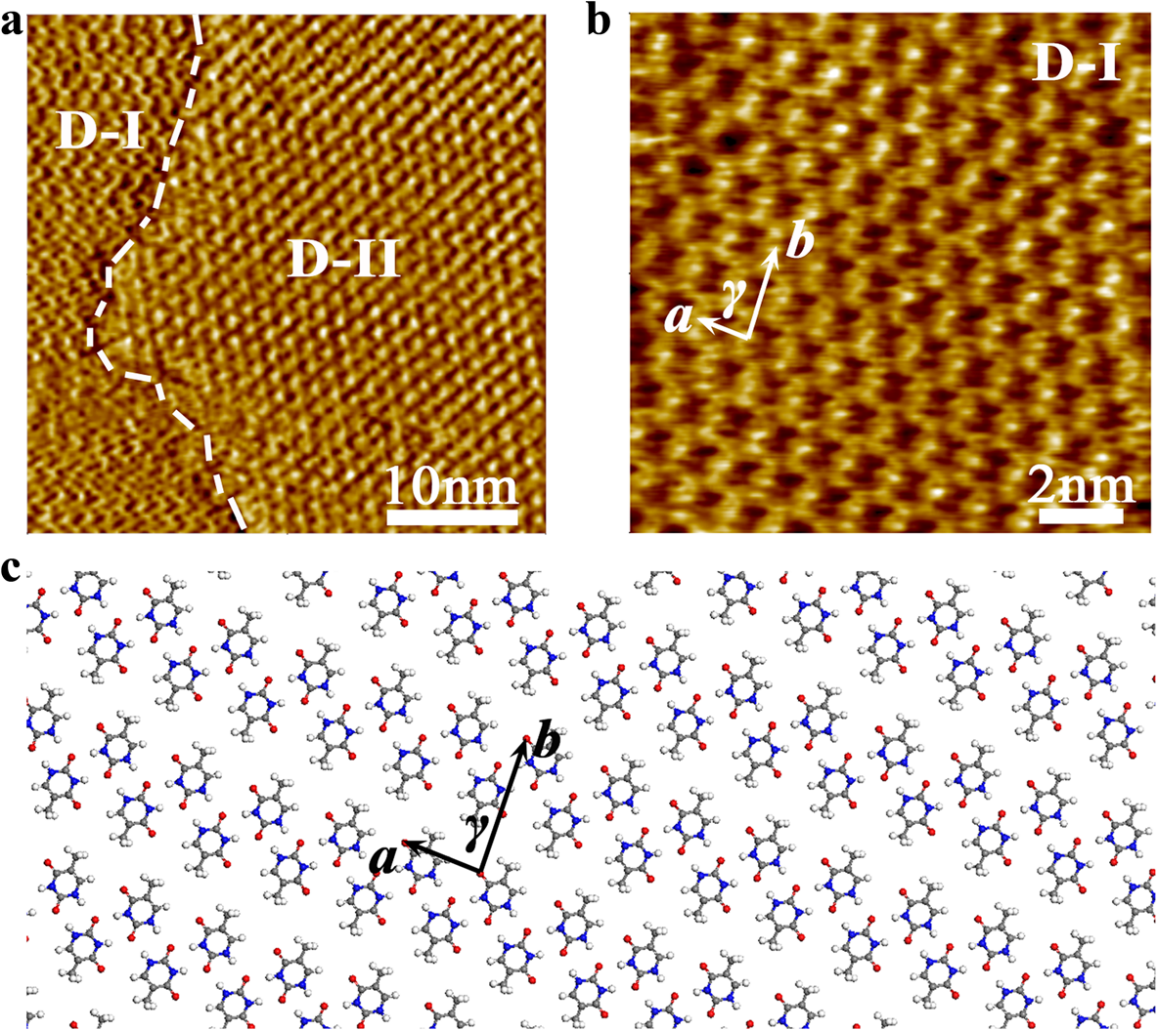
**a** STM image including two adjacent domains of the self-assembled products formed by melamine and thymine molecules on graphite surface. **b** HR-STM image for assembled structure in D-I domain formed by thymine molecules. Tunelling conditions: *V*_bias_ = 0.450 V, *I*_*t*_ = 0.102 nA. **c** Model proposed for the assembled structure formed by thymine molecules

After dropping the totally mixed solution composed of equal volume of melamine and thymine saturated solution onto the freshly cleaved graphite surface, ambient STM characterization was carried out with tip immersing into the solution. As the assembled film of pure melamine, there were many domains with obvious boundary formed by thymine-melamine binary system. In addition, these assembled morphologies of assembled regions are different and can be divided into two categories. Figure 3a is a typical STM image composed of two different self-assembly domains, and they were respectively marked with D-I and D-II. According to the assembled structure in Fig. 2, the pattern of D-II domain should be attributed to that formed by pure melamine. However, the assembled structure in D-I domain does not possess the hexagonal characteristics as that of D-II.

HR-STM image (Fig. 3b) can supply more detailed information about the assembled pattern in D-I domain, where the zigzag chainlike-shaped structure was formed. The parameters of the unit cell outlined in Fig. 2b are measured as *a* = 0.8 ± 0.2 nm, *b* = 1.5 ± 0.2 nm, and *γ* = 78 ± 2°, which are consistent to those of assembled structures formed by thymine molecules [33]. The molecular model was also given in Fig. 3c. It suggests that one thymine molecule interacts with neighboring molecules under the intermolecular non-covalent interaction of hydrogen bonding O···H−C, and then forms the interesting zigzag chainlike-shaped pattern on graphite surface. Obviously, the similar assembled structure of zigzag can be observed in D-I domain, meanwhile the characteristic porous network of melamine was existed in D-II area. Therefore, the phase separation phenomenon had happened when dropping thymine-melamine binary solution onto graphite surface.

Several repeated experiments were carried out in order to confirm this result. Actually, there is no hetero-compound composed of thymine and melamine observed. That is, the host porous network of melamine catching thymine guest molecule was not detected in our experiments. It might be explained that the molecular interactions among melamine molecules or among thymine molecules were more dominant than those of between melamine and thymine molecules. On the other hand, a methyl functional group in the chemical structural of thymine molecule might result in that it is difficult for thymine to insert into the porous network formed by melamine molecules.

### Co-Assembly of Cytosine-Melamine Binary System

**Fig. 4:**
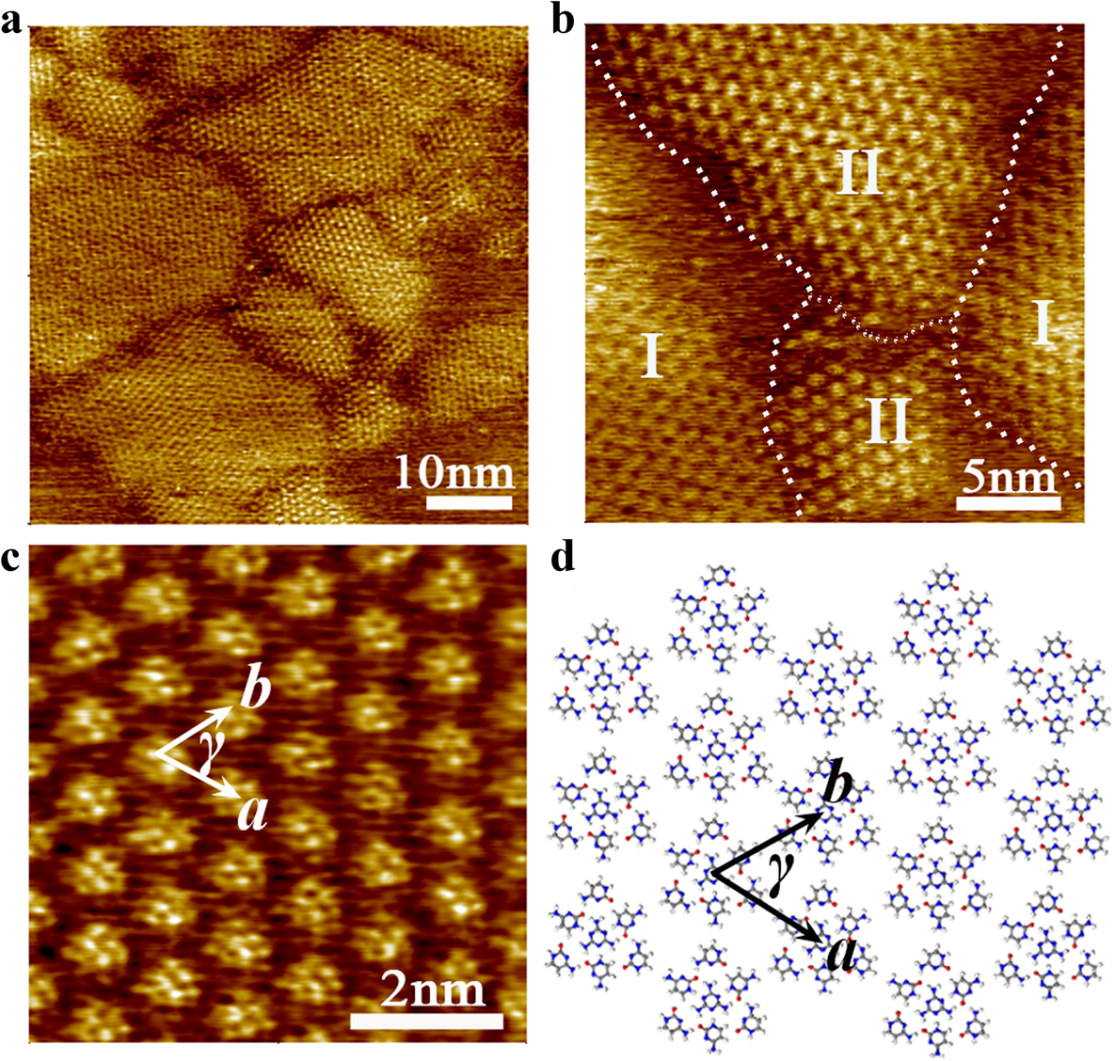
**a** A large-scale STM image showing multiple adjacent domains formed by cytosine-melamine binary assembly system on graphite surface. **b** HR-STM images including the typical adjacent domains marked with *I*, *II* where there existing different self-assembled units. **c** HR-STM image for the assembled structure in the domain marked with *II* in **b** image. Tunneling conditions: *V*_bias_ = 0.450 V, *I*_*t*_ = 0.189 nA. **d** Tentative molecular models proposed for the cytosine-melamine cluster configurations corresponding to **c**

Following the similar experimental procedure for investigating the co-assembly behavior of thymine-melamine binary system, cytosine-melamine binary system was used to study cytosine acting as guest molecule to the porous network of melamine. A series of STM images revealing the co-adsorption behavior of cytosine-melamine binary system, had been recorded under ambient conditions. Figure 4a gives a large-scale STM image including several molecular-assembled islands. These adjacent domains might be consisted of different components and different self-assembled structures, which can be further revealed by HR-STM images. One typical HR-STM image was given in Fig. 4b, where two distinct phases were marked with I, II in four domains. Compared with the assembled structure of melamine molecules, I-region exhibits the same hexagonal structure. However, the assembled unit of II-region magnified in Fig. 4c, was composed of several bright dots which were arranged as an interesting type of cluster-shaped structure. According to HR-STM images with molecular level resolution, it was revealed that this kind of assembled unit was more likely a distorted hexagonal rather than a six-fold symmetric structure.

The tentative molecular model (Fig. 4d) had been built and optimized by DMol3 package. In this hetero-component of cytosine and melamine, one melamine molecule is surrounded by six cytosine molecules. Three N atoms supplied by melamine molecule are connected to three H atoms devoted by six cytosine molecules via N···H−C hydrogen bonding, meanwhile every O atom of cytosine molecule is bonded with two H atoms contributed by one melamine and one cytosine to form O···H−C or O···H−N hydrogen bonds. Under the cooperation of these hydrogen bonding interactions, hetero-component as H-bonded functional molecular cluster was generated. The unit cells had been drawn in Fig. 4c and d, and they have the parameters of *a* = *b* = 1.3 ± 0.2 nm and *γ* = 57 ± 2°. Additionally, the desired host-guest structure of thymine-melamine binary system and the distinctive assembled rows of pure cytosine molecules [30] had not been detected, as the network formed by pure melamine molecules (I-region) as well.

## Discussion

In two binary assembly systems of thymine-melamine and cytosine-melamine, the self-assembled structures of melamine can be easily observed as like in the pure melamine assembly system. The reason should be that melamine molecule possesses a planar chemical structure with higher symmetry (*C*_*3*_), and its functional groups at three apexes construct numerous N···H−N intermolecular interaction among melamine molecules. Additionally, the aromatic-like core of melamine benefits van der Waals interaction happened between assembled molecule and graphite surface, which makes the self-assembled pattern of melamine formed easily. Therefore, the regular porous network formed by melamine molecules is availably fabricated under different experimental conditions, and their assembled architecture had been reported in many previous works. As for nucleic-acid bases, the lower structural symmetry and the weaker interactions of molecule-molecule and molecule-substrate are both not beneficial for the formation of assembled structures under ambient condition, especially where thermal fluctuation will affect the stability of the intermolecular interaction among cytosine molecules. Compared with cytosine, the chemical structure of thymine molecule has one more oxygen atom which increases the probability of forming hydrogen bonding between thymine molecules. This should be the reason why the self-assembled domain of pure thymine could be detected when investigating the thymine-melamine binary system. From these viewpoints, this work proves that molecular characteristics such as structural symmetry and functional groups play important roles during molecular self-assembly procedures.

**Fig. 5:**
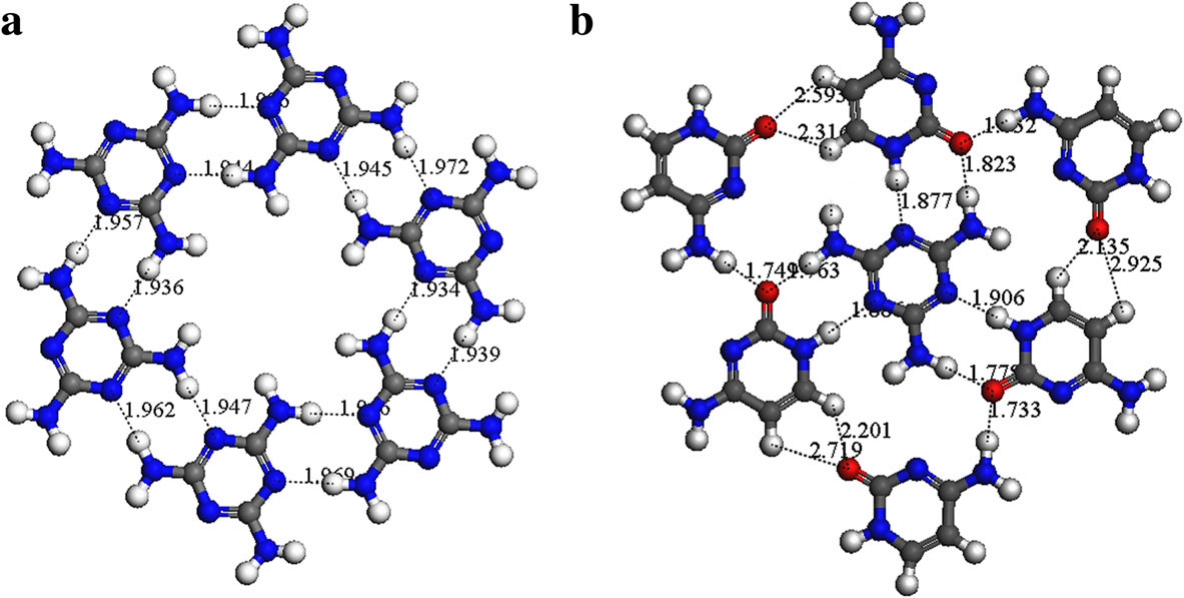
The DMol3 calculation results for pure melamine and cytosine-melamine binary assembly systems. **a** Melamine-melamine porous nanostructure. **b** One hetero-component molecular cluster of cytosine-melamine

However, being one of the favorable building blocks with planar structure, cytosine has been extensively studied in the past decades. Roberto et al. had reported that three kinds of motifs, zigzag filament, five- and six-membered rings could be constructed by cytosine molecules on Au(111) surface under UHV condition [38]. Xu et al. had revealed that the molecular features of pure cytosine molecules were arranged in molecular rows which could be assembled at 1-octanol/HOPG interface under ambient conditions [30]. However, there is no report about five- and six-membered rings of cytosine molecules found at liquid/solid interface. In this work, a new structure of molecular cluster composed of melamine and cytosine was detected when using cytosine-melamine binary system at 1-octanole/graphite interface. According to HR-STM image, their theoretical molecular model had been constructed and given in Fig. 4d. In order to explore the formation mechanism of this novel assembled unit, the detailed information of cytosine-melamine cluster was also given in Fig. 5b, as the ring-like structure of melamine in Fig. 5a as well.

In one single molecular cluster of cytosine-melamine, two kinds of hydrogen bonding exist. One is hydrogen bond of −C = O···H−N formed between cytosine and melamine, another one is hydrogen bond of −C = O···H−C among peripheral cytosine molecules. In Fig. 5b, the circumjacent six cytosine molecules were evenly divided into two types: three of them are bonded with other two neighboring cytosine molecule; the left three are also bonded with the central melamine molecule. The different bonding length and strength distorts the balance among them, and then leads to a lower symmetry of molecular cluster. In addition, N···H−N bond length of cytosine-melamine dimer with the help of −C = O···H hydrogen bonds is shorter than that in melamine-melamine dimer. Moreover, the bond length of −C = O···H-N in cytosine-melamine dimer distributes in a range from 0.18 nm to 0.19 nm. Usually, −C = O···H−N is stronger than N···H-N at an approximate hydrogen-bonding distance because the electronegativity of oxygen (O: 3.5) is more than that of nitrogen (N: 3.0) according to Pauling’s scale. Under three kinds of hydrogen-bonding interactions of N···H-N, −C = O···H−C and −C = O···H−N, molecules could regulate their self-assemble behavior to form a novel cluster-shaped molecular cluster in cytosine-melamine binary assembly system. The theoretical result in Additional file 1: Figure S1b, c also supports that, cytosine and melamine can form the steady structure of C−M dimer3 with binding energy ~ 0.70 eV through hydrogen bonds of N···H−N and −C = O···H−N existed in Fig. 5b. All in all, these experimental and theoretical results indicate that the characteristic advantages such as high symmetry, plane molecular structure, and abundant chemical groups, can benefit melamine molecules to form their unitary self-assembled architecture in both two binary assembly systems of thymine-melamine and cytosine-melamine. Especially, structure matching condition between building blocks plays a significant effect during the binary or heterogeneous assembly system.

## Conclusion

The co-assembly behaviors of melamine and two kinds of nucleic-acid bases (thymine and cytosine) were characterized by STM technique at liquid/solid (1-octanol/graphite) interface. The porous network of melamine, the zigzag chain of thymine, and the hetero-component cluster-like structure composed of cytosine and melamine, had been presented and discussed in detail. According to a series of experimental characterization and theoretical calculation, non-covalent intermolecular interaction of hydrogen bonding among molecules was the main driving force for the formation of these molecular assembled structures. This primary research provides some heuristic hints to fabricating the complex hetero-component materials composed of organic and biological molecules through employing self-assembly technique.

## Additional file

Additional file 1: Supporting information available. The optimized results of molecular dimers formed by three kinds of building blocks and the binding energies for molecular dimers. This information is available free of charge via the Internet or from the author. (DOCX 508 kb)

## Abbreviations

2D: Two-dimensional
DFT: Density functional theory
GGA: Generalized gradient approximation
HOPG: Highly oriented pyrolytic graphite
MPc: Metallophthalocyanines
PTCDI: Perylene-3,4,9,10-tetracarboxylic-3,4,9,10-diimide
STM: Scanning tunneling microscopy
sym-TTT: Symmetric triphenylene derivative with three carboxyl groups
TCDB: 1,3,5-tris(10-carboxydecyloxy)benzene
TMA: 1,3,5-benzenetricarboxylic acid, trimesic acid
UHV: Ultra-high vacuum
