# Supplementary material for: Investigating the Co-Adsorption Behavior of Nucleic-Acid Base (Thymine and Cytosine) and Melamine at Liquid/Solid Interface
Source: Nanoscale Res Lett. 2016 Dec 20;11:552. doi: 10.1186/s11671-016-1767-0 (PMC5174008; doi:10.1186/s11671-016-1767-0)

Supporting Information

Investigating the Co-adsorption Behaviour of Nucleic-acid-base (Thymine and Cytosine) and Melamine at Liquid/Solid Interface

*Huiling Zhao, Yinli Li, Dong Chen and Bo Liu**

Institute of Photo-biophysics, School of Physics and Electronics, Henan University, Kaifeng, 475004, P. R. China

*Corresponding Author.

E-mail: [zhl@henu.edu.cn](mailto:zhl@henu.edu.cn); [amlyl@henu.edu.cn](mailto:amlyl@henu.edu.cn); [dongchen@henu.edu.cn](mailto:dongchen@henu.edu.cn); [boliu@henu.edu.cn](mailto:boliu@henu.edu.cn)

**Fig. S1** (a) the chemical structures of building blocks (Melamine, M; Thymine, T; Cytosine, C); (b) the molecular dimers formed by melamine, thymine, and cytosine; (c) a table including the binding energies for the molecular dimers in (b).


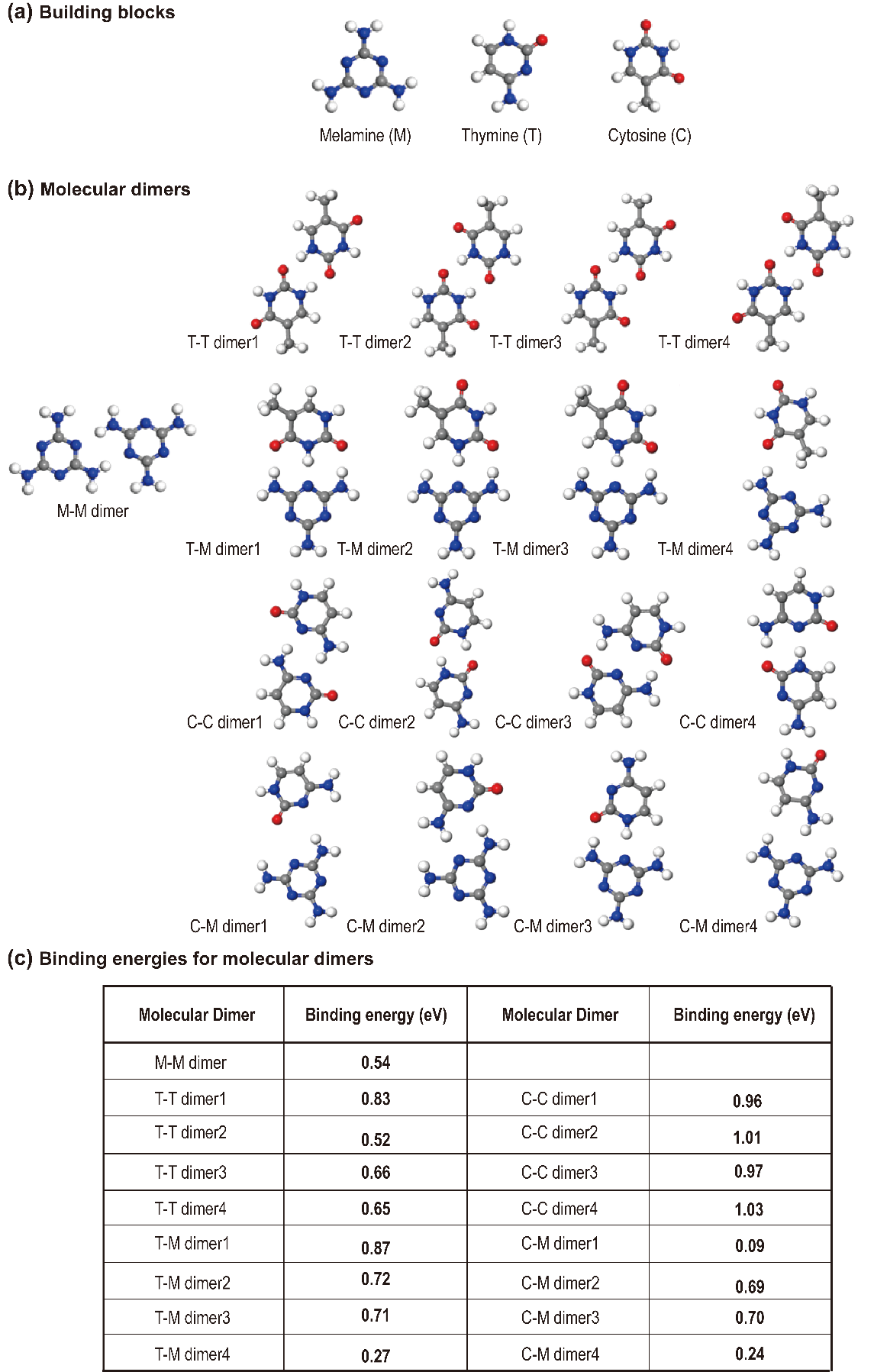


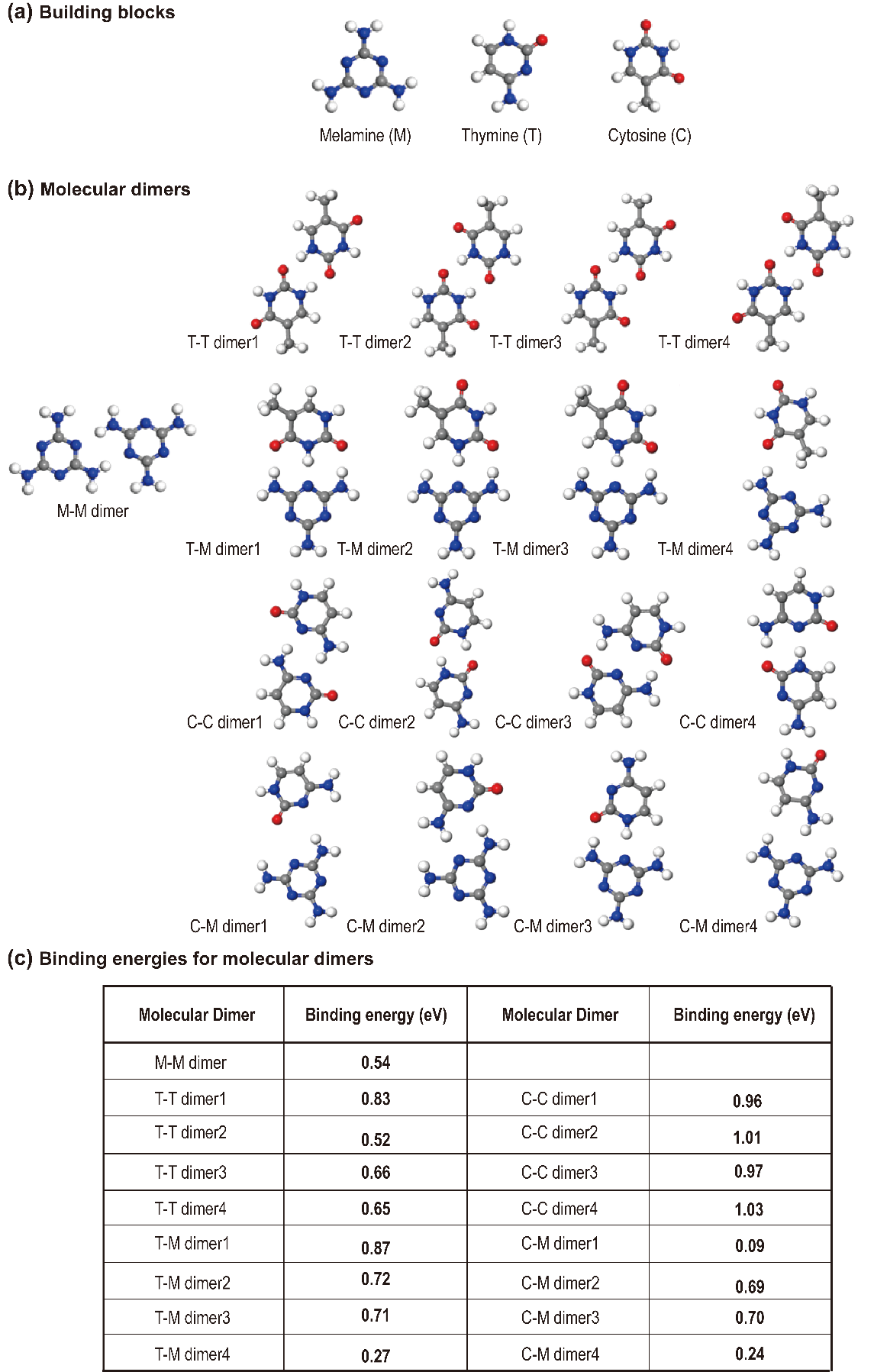

Supplement: Additional file 1: — Supporting information available. The optimized results of molecular dimers formed by three kinds of building blocks and the binding energies for molecular dimers. This information is available free of charge via the Internet or from the author. (DOCX 508 kb) [file 11671_2016_1767_MOESM1_ESM.docx]
